# Tackling the global impact of substandard and falsified and unregistered/unlicensed anti-tuberculosis medicines

**DOI:** 10.1177/23992026211070406

**Published:** 2022-01-23

**Authors:** Tamara Akpobolokemi, Rocio Teresa Martinez-Nunez, Bahijja Tolulope Raimi-Abraham

**Affiliations:** 1 Institute of Pharmaceutical Science, School of Cancer & Pharmaceutical Sciences, Faculty of Life Sciences & Medicine, King’s College London, London, UK; 2 Department of Infectious Diseases, School of Immunology & Microbial Sciences, Faculty of Life Sciences & Medicine, King’s College London, Guy’s Hospital, London, UK

**Keywords:** Substandard, falsified, unregistered, unlicensed, medicines, tuberculosis, drug resistance, antimicrobial resistance

## Abstract

Substandard and falsified (SF) medicines are a global health challenge with the World Health Organization (WHO) estimating that 1 in 10 of medicines in low- and middle-income countries (LMICs) are SF. Antimicrobials (i.e. antimalarials, antibiotics) are the most commonly reported SF medicines. SF medicines contribute significantly to the global burden of infectious diseases and antimicrobial resistance (AMR). This article discusses the challenges associated with the global impact of SF and unregistered/unlicensed antimicrobials with a focus on anti-TB medicines. Tuberculosis (TB) is the 13th leading cause of death worldwide, and is currently the second leading cause of death from a single infectious agent, ranking after COVID-19 and above HIV/AIDS. Specifically in the case of TB, poor quality of anti-TB medicines is among the drivers of the emergence of drug-resistant TB pathogens. In this article, we highlight and discuss challenges including the emergence of SF associated AMR, patient mistrust and lack of relevant data. We also present study reports to inform meaningful change. Recommended solutions involve the adaptation of interventions from high-income countries (HICs) to LMICS, the need for improvement in the uptake of medication authentication tools in LMICs, increased stewardship, and the need for global and regional multidisciplinary legal and policy cooperation, resulting in improved legal sanctions.

## Introduction

Tuberculosis (TB) is an infectious disease affecting both humans and animals. The causative agents of TB are *Mycobacterium tuberculosis* in humans, primates and guinea pigs and *Mycobacterium bovis* in cattle, rabbits and cats.^1^ Swine and dogs are susceptible to both *M. bovis* and *M. tuberculosis*.^1^ According to the World Health Organization (WHO) 2021 global TB report, TB killed about 1.3 million people.^2^ This is the second highest number of deaths resulting from a disease initiated by a single pathogen.^2^ Depending on the region, the prevalence of bovine TB can range from 0.1% to 50%; there were 140,000 new cases in 2019, with most animals slaughtered upon discovery of an infection.^3–7^

The increasing proximity between humans, livestock and wildlife, and its role in the transmission dynamics of mycobacterial infections, necessitates the need for a One Health approach in tackling TB infections. The One Health approach is an integrated multidisciplinary approach to attain optimal health for humans, animals and the environment in the surveillance of zoonotic diseases such as TB.^8^ The emergence of COVID-19 as a zoonotic disease,^9^ and its global burden in only 2 years (more than 260 million cases and nearly 5.2 million deaths as of 3 December 2021),^10^ must serve as a stellar warning for the urgent need for an integrative approach in tackling infectious diseases, with TB taking a toll of more than 1 million lives per year.

In humans, *M. tuberculosis* is spread through inhalation of small droplets from coughs or sneezes of an infected individual.^2,7^ ‘Latent’ TB infection and active TB disease are two subcategories of human TB. Latent TB is defined as a state of persistent immune response to stimulation by M. tuberculosis antigens without evidence of clinically manifested active TB.^11^ Active TB is characterised by the presence of TB disease as a result of *M. tuberculosis* infection^7^ and thus requires detection of the pathogen. TB disease is characterised by symptoms including a persistent cough, weight loss and night sweats.^12^ A quarter of the world’s population has latent TB infection with about 10% being at risk of progressing to active disease.^2^ In addition, it is estimated that every year 10 million people develop active TB disease.^2,7^ Eight low- and middle-income countries (LMICs) account for two-thirds of the global incidence of TB. Ranked from the highest to the lowest incidence rates, they include India, China, Indonesia, The Philippines, Pakistan, Nigeria, Bangladesh and South Africa.^2^

The WHO recommendation for newly diagnosed TB patients with no drug resistance involves two phases: the initial phase with the use of rifampicin, ethambutol hydrochloride, pyrazinamide and isoniazid (with pyridoxine hydrochloride), and then the continuation phase using the two drugs, rifampicin and isoniazid (with pyridoxine hydrochloride). It is important to note that the mentioned TB drugs (i.e. ethambutol, Isoniazid, rifampicin and pyrazinamide) are among the WHO’s list of essential medicines, prioritised for sufficient supply, affordability and access by the general population.^13^ The treatment regimen for drug-resistant TB varies depending on the type of resistance. In cases of isoniazid resistance TB, the treatment regime includes rifampicin, pyrazinamide, ethambutol and levofloxacin for 6 months and longer regimens are recommended in other forms of drug resistance.^14^

The WHO defines *substandard* (also referred to as out of specification) medicinal products as authorised medicinal products that have failed to pass their quality standards and/or specifications.^15,16^ *Falsified* medical products are products that have been intentionally mislabelled to misrepresent their composition or source.^15,16^ These are different to *unregistered or unlicensed* medical products, which are substances that are without approval and/or necessary evaluation by the regional or national regulatory authority for the market where they are used, distributed or sold^16^ Substandard and falsified (SF) medicinal products is the agreed simplified terminology used and will be used in this article moving forward. This commentary article focuses on the impact of anti-TB SF medicines, highlights their associated issues and offers solution-focused recommendations.

A total of 1 in 10 medicines in LMICs are SF.^17^ Anti-infective agents such as antimalarials (e.g. chloroquine, artemisinin-based combination therapies (ACTs)) and antibiotics such as anti TB medicines (e.g. isoniazid and rifampicin) are commonly reported as being SF.^17,18^ The main concern with SF anti-infectives is the impact of sub-therapeutic levels of the anti-infective (or their lack of effect) resulting in prolonged infection as well as contributing to antimicrobial resistance (AMR). Patients also have a false sense of security stemming from their expectation that the medical treatment received should work according to its intended purpose. It is important to note that heterogeneity does exist in this expectation, including patient location, confidence in healthcare institutions, mental and social conditioning.^19^

AMR is defined by microbial (bacteria, fungi, virus) changes that render medications ineffective and unable to cure infections.^20^ AMR is a serious global health issue and threat that prevents the effective treatment of microbial infections.^21^ Anti-infectives are to be used only when indicated, for the appropriate time and dose.^22^ This is the underlying principle of the globally accepted TB directly observed treatment (DOTS): to enable safe and reliable treatment against TB. Anti-TB SF drugs, therefore, undermine the DOTS standard.^23^ Their high representation among commonly SF drugs, similar to antimalarials, may also be reflected in TB drug resistance. The different forms of drug-resistant TB result in significantly longer treatment times and fatality rates of up to 80%,^24^ making TB a global threat prioritised by the WHO.^7,25^ In 2014, there were an estimated 700,000 deaths that occurred due to AMR and more than one-third of these were attributed to TB patients.^24^

Factors surrounding TB disease such as poor awareness and perception of the impact of the disease as well as associated mental health and psychiatric impacts make patients particularly vulnerable and more likely to be significantly impacted by anti-TB SF drugs. This is especially stark in low-resource regions situated in LMICs,^26,27^ which also have the highest rates of TB.^2^ As a comparison, studies on malaria perception have reported a significant awareness, even in low-resource settings, that the disease is caused by mosquitoes, dirty or stagnant water creates a breeding ground. Thus, mosquito nets, and insecticides are suitable preventive measures against malaria.^28–30^ In stark contrast to malaria, several studies have reported a gap in the depth of awareness for TB of what is necessary for infection evasion and preventability. Community beliefs that TB occurs due to bewitchment and curses have been reported in studies conducted in several other countries, including Ghana,^31^ Ethiopia,^32,33^ Rwanda^34^ and Uganda.^35^ Such beliefs have reported to impact marriage prospects, resulted in divorce, discrimination, forceful isolation of disease sufferers and withdrawal from society.^26,36^ These misconceptions have been shown to affect people’s perception of their risk as well as adherence to treatment or preventive measures.^26,37^ In addition, initial TB symptoms of cough and cold are often overlooked, and wrongly attributed to the common cold.^38^ This results in further delay in seeking appropriate healthcare and relying on self-medicating (mostly with traditional healers or local drug dispensaries) and in some cases until the individual’s health significantly deteriorates.^38,39^

TB patients have wrongly reported to have psychosocial and psychiatric disorders, most likely stemming from the poor perception of the disease, stigmatisation and even treatment-adverse reactions.^27,40–42^ Early researchers believed that the presence of mental illness creates a solid predisposition to TB and vice versa.^43^ Researchers and healthcare providers have reported various TB-associated psychiatric illnesses, including loss of interest in life, depression, psychosis, denial and anxiety.^27,44^ Poor perception, stigmatisation and TB psychopathology contribute to delay in seeking modern healthcare, loss of income and poor treatment adherence.^45–48^ The latter is a significant factor in the development of drug resistance.^49,50^ All these contribute to disease prevalence, increase patient expenses, and reduce the ability to obtain or perceived need for quality medicines or healthcare, making patients vulnerable to anti-TB SF medicines.^51,52^

## The emergence of drug resistance

Of concern is the contribution of SFs to AMR and overall impact on the prevalence of infectious diseases. Mutations are an inherent component of microorganismal proliferation^53–55^ as DNA replication during cell division can accumulate mutations, that is, changes in the nucleotide sequence. Polymerases have error rates, and thus with each round of replication mutations will accumulate.^54^ This may result in mutant generations with reduced susceptibility to medicines^56,57^ if those errors occur in processes that are the medicine’s targets. In a background of external pressure, such as that of low-efficient antimicrobial substances, mutant organisms that present a resistance gene will have a competitive advantage over those that do not have the specific mutation, resulting in reproductive competition ability that will affect drug susceptibility.^58^

**Figure 1.**
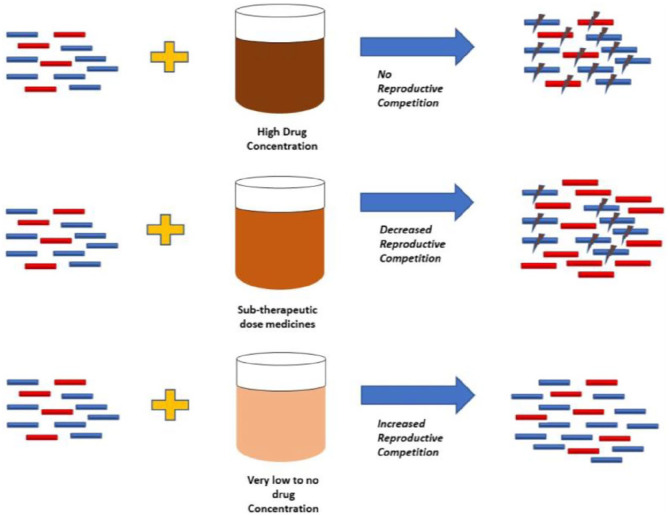
Development of drug resistance depending on drug concentration of active agent. Adapted from Pisani.^60^ Key: Drug – susceptible bacteria indicated by blue rods, resistant bacteria indicted by red rods. The lightning sign describes death.

High concentrations of the active pharmaceutical ingredient (API) during the early stages of infection kill susceptible microbes and may kill partially resistant mutants.^59^ However, SF medicines containing sub-therapeutic concentrations of the anti-infective may not be effective to achieve this, and thus mutant microorganisms will overproliferate while ‘wild type’ ones will perish.^59^ Therefore, resistant SFs (rSFs) can reduce the reproduction competition and allow for the proliferation of resistant microbes. This phenomenon is summarised in Figure 1.

There are two major forms of drug-resistant TB: multi-drug resistant TB (MDR-TB) and extensively drug-resistant TB (XDR-TB). MDR TB is defined as resistance to first-line TB medicines, that is, rifampicin and isoniazid.^61^ Both Isoniazid and rifampicin drug resistance arises due to mutations of genes encoding proteins that are essential in the targeted biological pathways. Resistance to isoniazid arises through bacterial mutations necessary for the blocking of the synthesis of mycolic acid activity such as *katG* and *inhA*.^62,63^ Conversely, resistance to rifampicin occurs through mutations of the *rpoB* gene which codes for M.TB RNA polymerase.^64^ XDR-TB occurs where MDR-TB is already present with additional resistance to fluoroquinolones and at least one of the three injectable second-line drugs, namely amikacin, kanamycin or capreomycin.^65^ Fluoroquinolone drug resistance can develop through mutations in the type II topoisomerase (DNA gyrase) gene.^66^ In addition, *M. tuberculosis* has also been shown to evolve active pumps against fluoroquinolones.^67^ Resistance to the second-line injectable drugs differs and is strongly dependent on their mechanism of action.^68–70^

Patients receiving lower therapeutic doses of anti-TB drugs are at risk of developing drug resistance and resultant treatment failure.^71,72^ Bate et al.^73^ performed quality control tests on 713 samples of isoniazid and rifampicin sourced from local pharmacies in 17 WHO regions, and found approximately 9% (65) of the samples were SF drugs with half of them possessing 10% to 80% isoniazid or rifampicin.

## Medicine costs and distrust

The economic impact and financial loss due to SF medicines in LMICs is estimated to be US$30.5 billion annually.^17^ This economic-financial cost includes several factors such as wasted human efforts and resources, economic loss for patients, their families and manufacturers of quality drugs.^17^ Patients lose income due to prolonged illness, with additional expenses incurred due to drug toxicity healthcare needs, failure of the treatment or even premature death.^17^

In LMICs, anti-TB SF drugs can contribute to an already existing loss of trust or confidence in the government and healthcare systems by the public.^74^ A 2011 systematic review by Berendes et al.^74^ suggested that poor perception of the health system, especially relating to the clinical skills and technical competence of clinicians, exists in LMICs. Qualitative research in China found that patients viewed the private health care system negatively, with references to ‘falsified doctors’ and ‘falsified drugs’.^75^ SF medicines are to worsen this already existing negative perception. Public health systems, including healthcare agencies and health practitioners, can also lose confidence about the effectiveness of TB medications, due to reports of drug resistance arising from SF drug use. As a result, pharmaceutical companies and health institutions invest in the research and discovery of new replacement agents. DiMasi et al.,^76^ showed that the cost of drug development grows faster than inflation by 7.4%. The estimated cost for the development of a single drug is US$2.6 billion.^77^ Even with high capital, antibiotics including anti-TB medicines offer reduced return on investment as their intake is limited, unlike other chronic disease drugs that are lifetime medications. Consequently, there is a low financial incentive as most of the consumers are in the poorest parts of the world.^24,78^

## Lack of documentation of rates or study reports on TB SFs

There is limited information on the actual adverse effects or death rates for TB due to SF medicines. This is in stark contrast to other diseases such as malaria or pneumonia. In sub-Saharan Africa, SF antimalarials are thought to cause 267,000 additional deaths, which is more than half of the total global malarial deaths per year.^17^ Taking into consideration the significantly higher fatality rates of TB (16%), compared with malaria (2%) and the already existing drug resistance issues, it appears safe to consider that SF drugs may be contributing to TB fatality more than believed.^79^ Anti-TB SF medicines containing rifampicin and pyrazinamide can contain excessive amounts of active agents, rather than the usual insufficient or absent active drug.^80^ Accidental overdose of TB drugs including rifampicin may cause severe adverse events (red man syndrome) or death.^81,82^ Unfortunately, there is a gap in the literature on the actual rates and incidence of excessive dosing by anti-TB SF medicines. This issue has also been highlighted by other studies.^83,84^ The WHO states an urgent need to obtain reliable data, particularly from sufficient sample sizes to aid reliability of the estimates of the impact and prevalence of anti-TB SF overdose.^17,83,84^

Several factors contribute to death by anti-TB SF medicines. First, there is invisibility that surrounds TB disease. When the disease affected high-income countries (HICs), it was at the forefront of public interest.^85,86^ However, due to better living conditions and access to quality healthcare, it seems like a forgotten plague.^87^ TB is now associated with poverty, affecting the most economically disadvantaged and people in low-resource rural regions.^88,89^ As such, although it continues to kill millions of people each year,^2,7^ TB remains invisible. Furthermore, the association with poverty, cost of diagnosis and treatment of TB necessitates improved access to anti-TB drugs in high-burden settings.^90–92^ Hence, the disease is heavily reliant on generic medicines.^90,93^ Ongoing debates on the negative impact of anti-counterfeiting measures on the (legitimate) generic medicines industry have caused distraction and resulted in delayed assessment of anti-TB SF-related issues.^94,95^ There is an urgent need for further rigorous in country-led research in anti-TB SF. Standardisation of medicine quality studies using methods such as the Medicine Quality Assessment Reporting Guidelines (MEDQUARG) may improve the quality of studies conducted and create homogeneousness.^96^ Theoretically, this approach could allow for comparability of studies across global regions. However, standardisation may also disadvantage regions that do not have the capacity (e.g. financial and logistical) nor consider or accommodate the heterogeneity within the individual regions. This will impact testing strategies, sampling and resource capacity.^97^ An updated guideline as suggested by McManus and Naughton,^97^ which allows the inclusion of contextual differences when reporting these medical quality studies, may help alleviate this issue.

## Recommended solutions

There is no one simple solution to the issue of SF drugs, specifically anti-TB SF medicines. Due to the limited data available as well as a few successful examples in the literature, some of the recommendations provided here are based on the author’s recommendations and not on reported work.

## Adaptation of interventions from HICs to LMICs

Although global harmonisation is constantly recommended, regional policy development and implementation may be more feasible and realistic. Policies and guidelines introduced at a global scale are recommended by HICs and this ‘one style fits all’ approach is not applicable in LMICs. This is confirmed by the difficulty faced by LMICs when it comes to implementing certain guidelines such as the WHO’s good manufacturing practices.^98^ Hence, other interventions must be appropriately adjusted and applied for implementation in LMICs. These include improvement of legal sanctions, increased stewardship, advocacy and regional cooperation in the implementation of international guidelines. The latter include medicine serialisation laws similar to the EU Falsified Medicines Directive (EU FMD) and The U.S. Drug Supply Chain Security Act (DSCSA).^99,100^ Medicines serialisation provides further data and detailed information on drug location and drug manufacturer throughout the supply chain.^101,102^ Adopting laws such as these will ensure manufacturers’ mandatory implementation of medicines serialisation technology and the traceability of drugs, especially in LMICs.^99,100^

It is important to note that drug authentication technologies, mainly through product verification, already exist in multiple LMICs, with apparent success. Notably, Nigeria’s National Agency for Food and Drug Administration and Control (NAFDAC) unique drug code allows the identification of legit drugs and has led to a significant reduction of counterfeit medicines from 40% in 2001 to 16.7% in 2015.^103^ However, there still exists a deficiency in data, visibility, distribution and illegitimate drug control gap that medicines serialisation technology can fill.^104^ These data can be leveraged to ascertain the specific location where an illegitimate drug entered the supply chain, allowing for precision and rapid recall. In addition, it can provide data on the incidence of falsified medicines, which is currently lacking and necessary for quantifying the problem, especially in LMICs.^105^ Hence, mandatory drug serialisation combined with authentication has the potential to bridge the gap of fake medicines detection standards of LMICs compared with HICs.

LMICs face different challenges in the implementation of serialisation technology. An important area is the scarcity of expertise and skilled personnel.^106^ Others include financials, logistics, technology/infrastructure, data management challenges and productivity/cost issues.^107^ Therefore to address these, actions such as investments in local skill development,^106^ either through know-how transfer by foreign experts/investors or skills training of existing workers, is essential. There needs to be a safe data infrastructure that allows for safe exchange of knowledge and tracking of medicines; while its management and access has to be local, it must allow for safe communication and exchange of information between regions and countries. In addition, fiscal or financial incentives can be introduced to allow sustainability and encourage implementation.^108^ For example, Turkey’s re-reimbursement of pharmacists for verified dispensing and prescriptions have been cited as significant to the success of the country’s drug track and trace system.^107,108^ Also, governmental investments in infrastructure such as ensuring optimal power and electricity supply to aid communications, digital operations and data management are necessary. Innovative approaches to this, including green and solar energy, may aid implementation, especially in low-resource settings.^109^ Finally, implementing a regional strategy where national hubs exist within individual countries. However, reporting from each region (e.g. West Africa) to a regional-level management authority^110^ may facilitate implementation in LMICs. This approach will enable the joining of resources by individual countries and reduce the burden of the challenges mentioned.

## Improved update of medication authentication tools in LMICs

Detection tools for infield testing of suspected SF drugs by evaluating API content are readily available.^111^ However, greater uptake and evaluation of usability in different settings in LMICs is required. For example, the technology firm Sproxil has taken a mass ‘track and trace’ or serialisation approach where a patient can scratch off a panel on the product at the point of purchase to reveal a code which is sent by text message to confirm whether the product is genuine.^112^ This approach has been effective with more than 28 million verifications and collaborations with multinationals such as GlaxoSmithKline.^113^ However, this solution will not work in settings where there is poor reception or poor understanding/trust on the technology itself (i.e. the person feeling that they are being tracked). With the impact of anti-TB SF medicines on TB drug level (i.e. sub-therapeutic levels resulting in drug resistance and excessive levels resulting in overdose with resultant adverse effects), implementing in field drug content testing as part of normal practice could prove beneficial. These approaches cannot be ‘one size fits all’ either, and must consider not only the technology itself but differences in culture, perception and feasibility of implementation. In some settings, having a person that ‘validates’ the purchased medicine may work better than allowing the users to do it themselves.

## Increased stewardship and advocacy

A multi-stakeholder advocacy and awareness strategy against the global impact of SF medicinal products is important. These can be done through television adverts, signages or posters providing palatable information for the public on how to identify SF medicines. This can then be specifically tailored for therapeutic agents of particular concern in the specific region, for example, anti-TB or antimalarial medicines in LMICs. SF medicines should be added as a specific area of advocacy in TB stewardship strategies. Importantly, awareness of SFs in the community can also start from school, so that children can learn that this exists and poses a real problem. There also exists a gap regarding the depth of knowledge of TB disease.^26,37^ As such, a different approach to awareness and education is important. Emphasis should be placed on disease education as it relates to the origin and causal agent of TB as well as better prognosis when modern healthcare services are utilised. This will make people better equipped at decision making regarding prevention, treatment and social measures. Understanding of TB, its consequences as well as the damage that anti-TB SF can cause are paramount for their rejection and reporting.

## Improvement of legal sanctions

Given that SF drugs cause financial loss, hospitalisation and fatalities, the penalties given should reflect these effects. The WHO has stressed that little or non-existent legislation for severe punishments for drug falsifying is a problem.^114^ Currently, the penalties for drug falsifiers in Nigeria is a fine of ₦500,000 (US$1200 (as of 2021)) and/or 5 to (*greater than*) 15 years in prison.^115^ Petty criminals see drug falsifying as a way of making a lot of money with lower penalties in comparison to cocaine, heroin or crack trade.^116^ Therefore, drug falsifiers gravitate towards countries with weak healthcare regulatory legislature and legal laws. Improving the legal sanctions, via regional and international cooperation, is paramount in stopping anti-TB SFs.

## Global and regional multidisciplinary legal and policy cooperation

An example of cooperation between different sectors has been seen in Rwanda where border customs and the ministry of health work together to ensure inspection of each shipment of TB drugs upon importation. Where SF drugs are found, they partner with the Rwandan police to charge and arrest the persons or criminals responsible.^117^ Rwanda’s TB rates have also significantly declined over the years and it recorded over 85% success rates in drug-resistant TB treatment.^118^ Nonetheless, the Rwandan government is aware of the necessity of working together with neighbouring countries. As such the Rwandan government and other East African countries have drafted a regional law of a unified plan against SF medicines.^117^ They also recently reviewed their laws to be in line with the African Union model law on medicine regulation which facilitates the harmonisation of efforts.^119^

## Conclusion

The problem of anti-TB SF medicines cannot be eradicated by the solo action of an institution or country, or individual stakeholders. Therefore, different sectors within and outside of countries or regions must cooperate and work together unanimously. The extent of damage SF medicine has on TB control needs to be acknowledged and reported. While there is information on the presence of anti-TB SF medicines in LMICs, there are no studies on the effect of SF drugs on resistance development problems and their contribution to the mortality rates of the disease. This is an area that requires urgent addressing to avoid further spread of resistant TB and to tackle the problem in these areas.

Anti-TB SF drugs are an economic and significant contributor to the persistence of the disease. It is time for the TB community to recognise and quantify the damage that is occurring, and to support the identification and implementation of interventions.
